# Supplementary material for: iDNA-ABF: multi-scale deep biological language learning model for the interpretable prediction of DNA methylations
Source: Genome Biol. 2022 Oct 17;23:219. doi: 10.1186/s13059-022-02780-1 (PMC9575223; doi:10.1186/s13059-022-02780-1)
Supplement: Supplementary file 1 — Additional file 1: Table S1. Performances of iDNA-ABF and the state-of-the-art methods on 17 benchmark datasets across species and methylation types. Table S2. Performance of our iDNA-ABF under various methylation patterns varied with different sequence lengths in three human cell lines. Table S3. Performance of our iDNA-ABF using ChIP-seq data varied with different sequence lengths in three human cell lines. Table S4. Performance of iDNA-ABF using ChIP-seq data + sequence data varied with different sequence lengths in three human cell lines. Table S5. The transfer learning performance of our model varied with different sequence lengths in three human cell lines. Table S6. The statistics of 17 benchmark datasets with three methylation types in various species. Table S7. The statistics of raw data with different methylation patterns in three human cell lines. Table S8. The statistics of the data in three human cell lines after sequence similarity reduction using CD-HIT. Table S9. The statistics of training and testing data under different methylation patterns in three human cell lines. Table S10. Performance comparison of different scales as the input to train the model in various species. Table S11. Performance comparison with the 5mC methods on cancer cell line Encyclopedia (CCLE). Table S12. Performance of iPromoter-5mC in three human cell lines. Table S13. Training parameters of our model on 17 benchmark datasets. Table S14. Performance of our iDNA-ABF for the SNP classification. Table S15. Performance of the multi-task model. Table S16. Performance of the regression model built on ChIP-seq data. Figure S1. The ROC curves on benchmark datasets. Figure S2. The PR curves on benchmark datasets. Figure S3. The UMAP visualization results on benchmark datasets. Figure S4. The SN, SP, and AUC of the models with and without adversarial training on 17 benchmark datasets with the independent test. Figure S5. Taxonomy tree and accuracy for eleven species in 6mA dataset. Fig [file 13059_2022_2780_MOESM1_ESM.pdf]

Additional file

Table S1. Performances of iDNA-ABF and the state-of-the-art methods on 17 benchmark datasets across species and methylation types.

Type	Dataset	Model	ACC	SN	SP	AUC	MCC
**5hmC**	*5hmC_H.sapiens*	iDNA_MS	0.9475	0.977	0.9181	0.9475	0.8966
		iDNA_ABT	0.9492	0.9863	0.9121	0.9492	0.9009
		iDNA_ABF	0.9501	0.9838	0.9164	0.9501	0.9022
		BERT6mA	0.9471	0.9761	0.9181	0.9471	0.8957
		Deep6mA	0.9317	0.9428	0.9206	0.9317	0.8636
		MM-6mAPred	0.9061	0.9752	0.837	0.9061	0.8201
	5hmC_M.musculus	iDNA_MS	0.9679	0.9685	0.9668	0.9679	0.9353
		iDNA_ABT	0.9685	0.9706	0.9663	0.9685	0.9369
		iDNA_ABF	0.9679	0.969	0.9668	0.9679	0.9358
		BERT6mA	0.9628	0.9587	0.9668	0.9628	0.9255
		Deep6mA	0.9597	0.97	0.9494	0.9597	0.9197
		MM-6mAPred	0.9307	0.9761	0.8852	0.9307	0.8649
**4mC**	*4mC_C.equisetifolia*	iDNA_MS	0.7109	0.7169	0.7049	0.7109	0.422
		iDNA_ABT	0.8251	0.7923	0.8579	0.8251	0.6517
		iDNA_ABF	0.8579	0.8743	0.8415	0.8579	0.7162
		BERT6mA	0.7732	0.8571	0.6557	0.7732	0.5622
		Deep6mA	0.7596	0.918	0.601	0.7596	0.5473
		MM-6mAPred	0.7732	0.683	0.8634	0.7732	0.5556
	4mC_F.vesca	iDNA_MS	0.8239	0.8297	0.8181	0.8239	0.648
		iDNA_ABT	0.842	0.852	0.8321	0.842	0.6842
		iDNA_ABF	0.8524	0.8535	0.8512	0.8524	0.7047
		BERT6mA	0.8221	0.9026	0.7416	0.8221	0.6527
		Deep6mA	0.8502	0.8672	0.8333	0.8502	0.7009
		MM-6mAPred	0.7639	0.7765	0.7513	0.7639	0.528
	4mC_S.cerevisiae	iDNA_MS	0.7042	0.7017	0.7068	0.7042	0.408
		iDNA_ABT	0.7027	0.6694	0.7361	0.7027	0.4064
		iDNA_ABF	0.723	0.6876	0.7583	0.723	0.447
		BERT6mA	0.6911	0.7128	0.6694	0.6911	0.3826
		Deep6mA	0.6871	0.727	0.6471	0.6871	0.3753
		MM-6mAPred	0.6891	0.6723	0.7057	0.6891	0.3783
	4mC_Tolypocladium	iDNA_MS	0.7115	0.7159	0.7076	0.7115	0.423
		iDNA_ABT	0.7383	0.7216	0.7549	0.7383	0.4768
		iDNA_ABF	0.7434	0.7385	0.7483	0.7434	0.4868
		BERT6mA	0.7354	0.7156	0.7552	0.7354	0.4712
		Deep6mA	0.7371	0.7536	0.7207	0.7371	0.4746
		MM-6mAPred	0.6695	0.6934	0.6457	0.6695	0.3396

**6mA**	*6mA_A.thaliana*	iDNA_MS	0.8377	0.8244	0.8511	0.8377	0.676
		iDNA_ABT	0.8538	0.8233	0.8842	0.8538	0.7088
		iDNA_ABF	0.8603	0.8264	0.8942	0.8603	0.7223
		BERT6mA	0.853	0.846	0.859	0.853	0.705
		Deep6mA	0.861	0.8451	0.877	0.861	0.7222
		MM-6mAPred	0.7553	0.7732	0.7373	0.7553	0.5109
	6mA_C.elegans	iDNA_MS	0.8557	0.8676	0.8437	0.8557	0.712
		iDNA_ABT	0.8903	0.8817	0.899	0.8903	0.7808
		iDNA_ABF	0.9138	0.9256	0.902	0.9138	0.8279
		Deep6mA	0.9018	0.9185	0.8851	0.9018	0.8042
		BERT6mA	0.902	0.908	0.895	0.902	0.803
		MM-6mAPred	0.7223	0.9052	0.5394	0.7223	0.4778
	6mA_C.equisetifolia	iDNA_MS	0.7113	0.7181	0.7046	0.7113	0.423
		iDNA_ABT	0.7328	0.6891	0.7765	0.7328	0.4673
		iDNA_ABF	0.7399	0.6713	0.8084	0.7399	0.4843
		BERT6mA	0.721	0.707	0.736	0.721	0.443
		Deep6mA	0.7288	0.6993	0.7583	0.7288	0.4584
		MM-6mAPred	0.6697	0.5944	0.7451	0.6697	0.3435
	6mA_D.melanogaster	iDNA_MS	0.8962	0.8897	0.9026	0.8962	0.792
		iDNA_ABT	0.9122	0.9038	0.9205	0.9122	0.8244
		iDNA_ABF	0.9228	0.9301	0.9155	0.9228	0.8457
		BERT6mA	0.8819	0.9378	0.8261	0.8819	0.7687
		Deep6mA	0.9199	0.9151	0.9247	0.9199	0.8398
		MM-6mAPred	0.809	0.8511	0.7669	0.809	0.6202
	6mA_F.vesca	iDNA_MS	0.9226	0.9394	0.9226	0.9226	0.846
		iDNA_ABT	0.9268	0.9233	0.9304	0.9268	0.8244
		iDNA_ABF	0.9413	0.9452	0.9375	0.9413	0.8827
		BERT6mA	0.926	0.925	0.926	0.926	0.851
		Deep6mA	0.9248	0.9413	0.9084	0.9248	0.8502
		MM-6mAPred	0.8507	0.9619	0.7395	0.8507	0.7195
	6mA_H.sapiens	iDNA_MS	0.8842	0.8631	0.9052	0.8842	0.769
		iDNA_ABT	0.898	0.894	0.902	0.898	0.796
		iDNA_ABF	0.9104	0.9057	0.9151	0.9104	0.8209
		BERT6mA	0.896	0.891	0.901	0.896	0.792
		Deep6mA	0.8987	0.9047	0.8926	0.8987	0.7974
		MM-6mAPred	0.8227	0.7978	0.8476	0.8227	0.6462
	6mA_R.chinensis	iDNA_MS	0.8545	0.8796	0.8294	0.8545	0.71
		iDNA_ABT	0.8261	0.8094	0.8428	0.8261	0.6525
		iDNA_ABF	0.8629	0.8328	0.893	0.8629	0.7271
		BERT6mA	0.781	0.743	0.819	0.781	0.564
		Deep6mA	0.8161	0.7859	0.8461	0.8161	0.6332
		MM-6mAPred	0.7859	0.8227	0.7491	0.7859	0.5734
	6mA_S.cerevisiae	iDNA_MS	0.7855	0.7538	0.8172	0.7855	0.572

		iDNA_ABT	0.8011	0.7237	0.8785	0.8011	0.6096
		iDNA_ABF	0.8278	0.7966	0.859	0.8278	0.6569
		BERT6mA	0.813	0.801	0.825	0.813	0.627
		Deep6mA	0.8011	0.7992	0.8029	0.8011	0.6022
		MM-6mAPred	0.7451	0.7279	0.7622	0.7451	0.4905
	6mA_T.thermophile	iDNA_MS	0.8563	0.9579	0.7548	0.8563	0.728
		iDNA_ABT	0.874	0.9334	0.8154	0.874	0.754
		iDNA_ABF	0.8804	0.9442	0.8167	0.8804	0.7671
		BERT6mA	0.874	0.925	0.823	0.874	0.752
		Deep6mA	0.8716	0.9343	0.8118	0.8716	0.7547
		MM-6mAPred	0.7474	0.9216	0.5732	0.7474	0.5958
	6mA_Tolypocladium	iDNA_MS	0.7342	0.7425	0.7259	0.7342	0.468
		iDNA_ABT	0.7738	0.7176	0.8301	0.7738	0.5512
		iDNA_ABF	0.7771	0.7649	0.7892	0.7771	0.5543
		BERT6mA	0.752	0.772	0.732	0.752	0.505
		Deep6mA	0.7619	0.7359	0.788	0.7619	0.5246
		MM-6mAPred	0.7051	0.5677	0.8425	0.7051	0.4267
	6mA_Xoc BLS256	iDNA_MS	0.8451	0.825	0.8652	0.8451	0.691
		iDNA_ABT	0.8694	0.889	0.8492	0.8694	0.7394
		iDNA_ABF	0.8817	0.8808	0.8827	0.8817	0.7634
		BERT6mA	0.8633	0.848	0.878	0.8633	0.7266
		Deep6mA	0.8421	0.8234	0.8321	0.8421	0.7423
		MM-6mAPred	0.7507	0.6997	0.8044	0.7507	0.5061

Table S2. Performance of our iDNA-ABF under various methylation patterns varied with different sequence lengths in three human cell lines.

Cell Lines	Pattern		ACC	SN	SP	AUC	MCC
**GM12878**	CPG	11bp	0.5358	0.3983	0.6733	0.5358	0.074
		41bp	0.7498	0.8428	0.6567	0.7498	0.5084
		71bp	0.7604	0.8888	0.6321	0.7604	0.539
		101bp	0.7575	0.9452	0.5697	0.7575	0.5556
	CHG	11bp	—	—	—	—	—
		41bp	0.7577	0.8533	0.6622	0.7577	0.5252
		71bp	0.7366	0.82	0.6533	0.7366	0.48
		101bp	0.6927	0.7888	0.5966	0.6927	0.3928
	CHH	11bp	—	—	—	—	—
		41bp	0.7845	0.835	0.734	0.7845	0.5719
		71bp	0.83	0.8736	0.7863	0.83	0.6625
		101bp	0.7775	0.9303	0.6247	0.7775	0.5829
**K562**	CPG	11bp	0.5243	0.3953	0.6533	0.5243	0.05
		41bp	0.7377	0.8353	0.6402	0.7377	0.4847

		71bp	0.7496	0.8375	0.6617	0.7496	0.5071
		101bp	0.7476	0.8838	0.6113	0.7476	0.5146
	CHG	11bp	—	—	—	—	—
		41bp	0.67	0.806	0.5333	0.67	0.353
		71bp	0.668	0.61	0.726	0.668	0.338
		101bp	0.666	0.6166	0.7166	0.666	0.335
	CHH	11bp	—	—	—	—	—
		41bp	0.728	0.834	0.622	0.728	0.4666
		71bp	0.767	0.806	0.728	0.767	0.5356
		101bp	0.681	0.74	0.622	0.681	0.3645
**HepG2**	CPG	11bp	0.5379	0.4736	0.6022	0.5379	0.076
		41bp	0.8336	0.8818	0.7853	0.8336	0.6702
		71bp	0.8579	0.8994	0.8165	0.8579	0.7184
		101bp	0.8557	0.8941	0.8173	0.8557	0.7135
	CHG	11bp	—	—	—	—	—
		41bp	0.7875	0.785	0.79	0.7875	0.575
		71bp	0.7962	0.86	0.7325	0.7962	0.5973
		101bp	0.8387	0.9125	0.765	0.8387	0.6849
	CHH	11bp	—	—	—	—	—
		41bp	0.7945	0.876	0.713	0.7945	0.5969
		71bp	0.7955	0.856	0.735	0.7955	0.5953
		101bp	0.838	0.925	0.751	0.838	0.6864

Table S3. Performance of our iDNA-ABF using ChIP-seq data varied with different sequence lengths in three human cell lines.

Cell Lines		ACC	SN	SP	AUC	MCC
**GM12878**	11bp	0.7	0.877	0.5231	0.7	0.4277
	41bp	0.683	0.7693	0.5967	0.683	0.3716
	71bp	0.6867	0.8021	0.5713	0.6867	0.3838
	101bp	0.6848	0.7968	0.5728	0.6848	0.3792
**K562**	11bp	0.71255	0.71255	0.71255	0.71255	0.71255
	41bp	0.714	0.714	0.714	0.714	0.714
	71bp	0.7113	0.7113	0.7113	0.7113	0.7113
	101bp	0.7099	0.7099	0.7099	0.7099	0.7099
**HepG2**	11bp	0.6529	0.6453	0.6605	0.6529	0.3058
	41bp	0.6499	0.7116	0.5883	0.6499	0.3022
	71bp	0.6449	0.6852	0.6046	0.6449	0.2908
	101bp	0.643	0.6946	0.5914	0.643	0.2875

Table S4. Performance of iDNA-ABF using ChIP-seq data + sequence data varied

with different sequence lengths in three human cell lines.

Cell Lines		ACC	SN	SP	AUC	MCC
GM12878	11bp	0.6433	0.5211	0.7655	0.6433	0.2956
	41bp	0.7966	0.8572	0.7361	0.7966	0.5977
	71bp	0.8207	0.8696	0.7718	0.8207	0.6445
	101bp	0.8149	0.9276	0.7023	0.8149	0.6465
K562	11bp	0.6866	0.6271	0.7461	0.6866	0.3759
	41bp	0.8055	0.8538	0.7572	0.8055	0.6139
	71bp	0.8117	0.8701	0.7533	0.8117	0.6278
	101bp	0.798	0.8404	0.7556	0.798	0.5982
HepG2	11bp	0.7731	0.9938	0.5524	0.7731	0.6087
	41bp	0.8418	0.8761	0.8075	0.8418	0.6852
	71bp	0.87	0.9042	0.8357	0.87	0.7416
	101bp	0.8833	0.9148	0.8518	0.8833	0.7681

Table S5. The transfer learning performance of our model varied with different sequence lengths in three human cell lines.

Cell Lines	Pattern		ACC	SN	SP	AUC	MCC
GM12878	CHG	11bp	—	—	—	—	—
		41bp	0.7611	0.8522	0.67	0.7611	0.5311
		71bp	0.7611	0.8533	0.6688	0.7611	0.5313
		101bp	0.7544	0.8666	0.6422	0.7544	0.5222
	CHH	11bp	—	—	—	—	—
		41bp	0.8202	0.856	0.7843	0.8202	0.642
		71bp	0.8412	0.894	0.7883	0.8412	0.6862
		101bp	0.836	0.9027	0.7693	0.836	0.6781
K562	CHG	11bp	—	—	—	—	—
		41bp	0.7366	0.7566	0.7166	0.7366	0.4737
		71bp	0.7483	0.85	0.6466	0.7483	0.5072
		101bp	0.7316	0.7733	0.69	0.7316	0.4649
	CHH	11bp	—	—	—	—	—
		41bp	0.738	0.728	0.748	0.738	0.476
		71bp	0.795	0.854	0.736	0.795	0.5941
		101bp	0.781	0.8	0.762	0.781	0.5624
HepG2	CHG	11bp	—	—	—	—	—
		41bp	0.825	0.8525	0.7975	0.825	0.6509
		71bp	0.83375	0.84	0.8275	0.83375	0.6675
		101bp	0.85625	0.925	0.7875	0.85625	0.7193
	CHH	11bp	—	—	—	—	—
		41bp	0.8385	0.812	0.865	0.8385	0.6779

		71bp	0.828	0.878	0.778	0.828	0.6593
		101bp	0.869	0.903	0.835	0.869	0.7397

Table S6. The statistics of 17 benchmark datasets with three methylation types in various species.

species	5hmc				6mA				4mC			
	Traning data		Testing data		Traning data		Testing data		Traning data		Testing data	
	pos	neg	pos	neg	pos	neg	pos	neg	pos	neg	pos	neg
A.thaliana	—	—	—	—	15937	15937	15936	15936	—	—	—	—
C.elegans	—	—	—	—	3981	3981	3980	3980	—	—	—	—
C.equisetifolia	—	—	—	—	3033	3033	3033	3033	366	366	366	366
D.melanogaster	—	—	—	—	5596	5596	5595	5595	—	—	—	—
F.vesca	—	—	—	—	1551	1551	1551	1551	7899	7899	7898	7898
H.sapiens	1172	1172	1172	1172	9168	9168	9167	9167	—	—	—	—
M.musculus	1840	1840	1839	1839	—	—	—	—	—	—	—	—
R.chinensis	—	—	—	—	300	300	300	300	—	—	—	—
S.cerevisiae	—	—	—	—	1893	1893	1893	1893	990	990	989	989
Tolypocladium	—	—	—	—	1690	1690	1689	1689	7664	7664	7663	7663
T.thermophile	—	—	—	—	29819	37184	53800	53800	—	—	—	—
Xoc.BLS256	—	—	—	—	8608	8608	8607	8607	—	—	—	—

Table S7. The statistics of raw data with different methylation patterns in three human cell lines.

Cell Lines		CpG	CHG	CHH
**GM12878**	Positive	2351130	2204	9948
	Negative	698867	1939543	9706039
**K562**	Positive	914525	775	2017
	Negative	666689	1875847	6264385
**HepG2**	Positive	1101829	1165	2499
	Negative	1047914	2436958	8468416

Table S8. The statistics of the data in three human cell lines after sequence similarity reduction using CD-HIT.

Cell Lines		CpG		CHG		CHH	
		pos	neg	pos	neg	pos	neg
GM12878	11bp	44736	34255	1448	30866	6022	155742
	41bp	1520062	285830	1919	724029	8402	637816
	71bp	3352912	268712	1972	670192	8868	589226
	101bp	1734150	254661	2007	613172	9046	543887
K562	11bp	39613	34255	550	30664	1439	68205
	41bp	554571	270206	671	694056	1717	547409
	71bp	627744	255023	691	644811	1800	501730
	101bp	659102	242096	711	589883	1836	458113
HepG2	11bp	41088	37962	717	32675	1802	85468
	41bp	667634	528172	924	1211241	2103	645473
	71bp	745767	488408	965	1095370	2238	613410
	101bp	785858	458661	1006	996748	2301	578197

Table S9. The statistics of training and testing data under different methylation patterns in three human cell lines.

Cell Lines		CpG				CHG				CHH			
		Traning data		Testing data		Traning data		Testing data		Traning data		Testing data	
		pos	neg	pos	neg	pos	neg	pos	neg	pos	neg	pos	neg
GM12878	11bp	20000	20000	10000	10000	500	500	500	500	5000	5000	1000	1000
	41bp	150000	150000	30000	30000	1000	1000	900	900	5000	5000	3000	3000
	71bp	150000	150000	30000	30000	1000	1000	900	900	5000	5000	3000	3000
	101bp	150000	150000	30000	30000	1000	1000	900	900	5000	5000	3000	3000
K562	11bp	20000	20000	10000	10000	300	300	200	200	1000	1000	4000	4000
	41bp	150000	150000	30000	30000	300	300	300	300	1000	1000	5000	5000
	71bp	150000	150000	30000	30000	300	300	300	300	1000	1000	5000	5000
	101bp	150000	150000	30000	30000	300	300	300	300	1000	1000	5000	5000
HepG2	11bp	20000	20000	10000	10000	500	500	200	200	1000	1000	800	800
	41bp	400000	400000	20000	20000	500	500	400	400	1000	1000	1000	1000
	71bp	400000	400000	20000	20000	500	500	400	400	1000	1000	1000	1000
	101bp	400000	400000	20000	20000	500	500	400	400	1000	1000	1000	1000

Table S10. Performance comparison of different scales as the input to train the model in various species.

Class	Dataset	Model	ACC	SN	SP	AUC	MCC
**5hmC**	*5hmC_H.sapiens*	3-mer	0.9501	0.9821	0.9181	0.9681	0.902

		4-mer	0.9497	0.9821	0.9172	0.9658	0.9012
		5-mer	0.9488	0.9812	0.9164	0.9677	0.8995
		6-mer	0.9475	0.977	0.9181	0.9647	0.8966
		3-4mer	0.9462	0.977	0.9155	0.9553	0.8942
		3-5mer	0.9497	0.9812	0.9181	0.9181	0.9011
		3-6mer	0.9501	0.9838	0.9164	0.9675	0.9022
		4-5mer	0.9475	0.9778	0.9172	0.9472	0.8967
		4-6mer	0.948	0.977	0.9189	0.9673	0.8974
		5-6mer	0.9467	0.9778	0.9155	0.9604	0.8951
	5hmC_M.musculus	3-mer	0.9676	0.969	0.9663	0.9821	0.9353
		4-mer	0.9668	0.969	0.9647	0.9836	0.9337
		5-mer	0.9668	0.969	0.9647	0.9841	0.9337
		6-mer	0.9652	0.9695	0.9608	0.9823	0.9304
		3-4mer	0.9644	0.969	0.9598	0.9783	0.9288
		3-5mer	0.9676	0.9695	0.9657	0.9828	0.9353
		3-6mer	0.9679	0.969	0.9668	0.9791	0.9358
		4-5mer	0.9657	0.9685	0.963	0.9775	0.9315
		4-6mer	0.9679	0.969	0.9668	0.976	0.9358
		5-6mer	0.966	0.969	0.963	0.9834	0.932
**4mC**	*4mC_C.equisetifolia*	3-mer	0.8306	0.8361	0.8251	0.872	0.6612
		4-mer	0.8115	0.7978	0.8251	0.8827	0.6232
		5-mer	0.8607	0.9071	0.8142	0.918	0.7244
		6-mer	0.8333	0.8033	0.8634	0.8737	0.6679
		3-4mer	0.8333	0.847	0.8197	0.9046	0.6669
		3-5mer	0.8361	0.8525	0.8197	0.9038	0.6725
		3-6mer	0.8579	0.8743	0.8415	0.9089	0.7162
		4-5mer	0.8743	0.8798	0.8689	0.9228	0.7487
		4-6mer	0.8306	0.8033	0.8579	0.8778	0.6622
		5-6mer	0.8525	0.8033	0.9016	0.9086	0.7084
	4mC_F.vesca	3-mer	0.8492	0.8873	0.8111	0.9226	0.7004
		4-mer	0.8383	0.8412	0.8354	0.9127	0.6766
		5-mer	0.8458	0.8939	0.7978	0.9189	0.6949
		6-mer	0.8427	0.8848	0.8006	0.9182	0.6878
		3-4mer	0.8418	0.8797	0.8039	0.9153	0.6856
		3-5mer	0.8503	0.8674	0.8331	0.9246	0.701
		3-6mer	0.8524	0.8535	0.8512	0.9282	0.7047
		4-5mer	0.85	0.8529	0.847	0.9254	0.6999
		4-6mer	0.8496	0.8876	0.8116	0.9251	0.7012
		5-6mer	0.8461	0.8571	0.8351	0.921	0.6924
	4mC_S.cerevisiae	3-mer	0.6582	0.8069	0.5096	0.7348	0.3315
		4-mer	0.7123	0.7189	0.7058	0.77	0.4247
		5-mer	0.7184	0.726	0.7108	0.7763	0.4369
		6-mer	0.6795	0.6562	0.7027	0.7323	0.3593

		3-4mer	0.7103	0.6785	0.7422	0.7666	0.4215
		3-5mer	0.686	0.7725	0.5996	0.7534	0.3778
		3-6mer	0.723	0.6876	0.7583	0.7897	0.447
		4-5mer	0.7265	0.7017	0.7513	0.792	0.4535
		4-6mer	0.7088	0.6269	0.7907	0.7828	0.4233
		5-6mer	0.6888	0.6169	0.7807	0.7728	0.4133
	4mC_Tolypocladium	3-mer	0.7359	0.7136	0.7523	0.8159	0.4662
		4-mer	0.738	0.6674	0.7523	0.8059	0.4662
		5-mer	0.7342	0.7467	0.7216	0.8092	0.4685
		6-mer	0.7329	0.7136	0.7523	0.8059	0.4662
		3-4mer	0.7283	0.7167	0.7399	0.7997	0.4567
		3-5mer	0.7314	0.7637	0.6992	0.4638	0.8087
		3-6mer	0.7434	0.7385	0.7483	0.8213	0.4868
		4-5mer	0.7313	0.7094	0.7532	0.4631	0.8067
		4-6mer	0.7419	0.7497	0.734	0.8181	0.4838
		5-6mer	0.7357	0.7266	0.7447	0.8149	0.4714
**6mA**	*6mA_A.thaliana*	3-mer	0.8552	0.8299	0.8805	0.9285	0.7113
		4-mer	0.8553	0.8263	0.8842	0.9288	0.7117
		5-mer	0.8555	0.812	0.899	0.9311	0.7137
		6-mer	0.8594	0.8311	0.8877	0.9326	0.72
		3-4mer	0.8572	0.8233	0.8911	0.9302	0.7161
		3-5mer	0.8555	0.8368	0.8742	0.929	0.7115
		3-6mer	0.8603	0.8264	0.8942	0.9349	0.7223
		4-5mer	0.8541	0.8478	0.8605	0.9288	0.7083
		4-6mer	0.8616	0.8424	0.8808	0.9341	0.7237
		5-6mer	0.8592	0.8154	0.9029	0.9331	0.7211
	6mA_C.elegans	3-mer	0.9029	0.9241	0.8817	0.9642	0.8065
		4-mer	0.9063	0.9299	0.8827	0.9649	0.8135
		5-mer	0.8662	0.8686	0.8638	0.9404	0.7324
		6-mer	0.9084	0.9259	0.891	0.964	0.8173
		3-4mer	0.899	0.9035	0.8945	0.9599	0.798
		3-5mer	0.8997	0.9171	0.8824	0.9618	0.8
		3-6mer	0.9138	0.9256	0.902	0.9682	0.8279
		4-5mer	0.9077	0.8807	0.9347	0.9677	0.8165
		4-6mer	0.9093	0.9201	0.8985	0.9641	0.8188
		5-6mer	0.9177	0.9369	0.8985	0.9696	0.836
	6mA_C.equisetifolia	3-mer	0.7191	0.6924	0.7458	0.7915	0.4388
		4-mer	0.7221	0.7491	0.695	0.7943	0.4448
		5-mer	0.6965	0.638	0.755	0.7617	0.3957
		6-mer	0.7324	0.7273	0.7376	0.8118	0.4649
		3-4mer	0.7275	0.7105	0.7445	0.8038	0.4553
		3-5mer	0.7047	0.7471	0.6624	0.7829	0.411

		3-6mer	0.7399	0.6713	0.8084	0.8098	0.4843
		4-5mer	0.7242	0.6644	0.784	0.7963	0.4516
		4-6mer	0.7369	0.7633	0.7105	0.8188	0.4744
		5-6mer	0.738	0.6957	0.7804	0.8135	0.4778
	6mA_D.melanogaster	3-mer	0.9177	0.9176	0.9178	0.9671	0.8354
		4-mer	0.9134	0.9135	0.9133	0.9638	0.8268
		5-mer	0.9131	0.9185	0.9078	0.9652	0.8263
		6-mer	0.922	0.8996	0.9444	0.9711	0.8448
		3-4mer	0.9135	0.9164	0.9106	0.9644	0.827
		3-5mer	0.9195	0.9303	0.9087	0.9693	0.8392
		3-6mer	0.9228	0.9301	0.9155	0.9713	0.8457
		4-5mer	0.9148	0.9028	0.9269	0.9648	0.8299
		4-6mer	0.9231	0.934	0.9121	0.9704	0.8463
		5-6mer	0.9217	0.9267	0.9167	0.9709	0.8435
	6mA_F.vesca	3-mer	0.9288	0.9336	0.9239	0.9723	0.8576
		4-mer	0.9352	0.9239	0.9465	0.9782	0.8706
		5-mer	0.9149	0.9155	0.9142	0.9698	0.8298
		6-mer	0.9371	0.9259	0.9484	0.979	0.8745
		3-4mer	0.931	0.9246	0.9375	0.9764	0.8621
		3-5mer	0.9371	0.931	0.9433	0.9774	0.8743
		3-6mer	0.9413	0.9452	0.9375	0.9804	0.8827
		4-5mer	0.9362	0.9426	0.9297	0.9767	0.8724
		4-6mer	0.9362	0.9381	0.9342	0.9801	0.8723
		5-6mer	0.9371	0.9529	0.9213	0.9807	0.8747
	6mA_H.sapiens	3-mer	0.904	0.9042	0.9038	0.966	0.808
		4-mer	0.902	0.8973	0.9067	0.9654	0.8041
		5-mer	0.9023	0.9003	0.9042	0.9653	0.8045
		6-mer	0.9039	0.9062	0.9016	0.9645	0.8078
		3-4mer	0.9044	0.8891	0.9198	0.9667	0.8093
		3-5mer	0.9038	0.8867	0.921	0.9684	0.8082
		3-6mer	0.9104	0.9057	0.9151	0.9695	0.8209
		4-5mer	0.8988	0.9096	0.888	0.9652	0.7977
		4-6mer	0.9076	0.8985	0.9167	0.9675	0.8153
		5-6mer	0.9081	0.9147	0.9016	0.9705	0.8164
	6mA_R.chinensis	3-mer	0.6756	0.4883	0.8629	0.7651	0.3787
		4-mer	0.7441	0.7726	0.7157	0.8165	0.4891
		5-mer	0.8545	0.8261	0.8829	0.9263	0.7102
		6-mer	0.8261	0.8896	0.7625	0.9189	0.6575
		3-4mer	0.7057	0.7023	0.709	0.764	0.4114
		3-5mer	0.6973	0.5217	0.8729	0.7877	0.4215
		3-6mer	0.8629	0.8328	0.893	0.931	0.7271
		4-5mer	0.8261	0.8027	0.8495	0.8944	0.6529
		4-6mer	0.8278	0.8528	0.8027	0.8869	0.6563

		5-6mer	0.8478	0.8595	0.8361	0.9042	0.6958
	6mA_S.cerevisiae	3-mer	0.7831	0.6587	0.9076	0.8636	0.5847
		4-mer	0.8146	0.794	0.8352	0.8914	0.6297
		5-mer	0.7826	0.7781	0.7871	0.8622	0.5653
		6-mer	0.8241	0.8135	0.8347	0.9006	0.6483
		3-4mer	0.8196	0.7771	0.8621	0.8969	0.6415
		3-5mer	0.8093	0.7655	0.8531	0.8874	0.621
		3-6mer	0.8278	0.7966	0.859	0.9062	0.6569
		4-5mer	0.7882	0.6962	0.8801	0.8653	0.5863
		4-6mer	0.8222	0.8193	0.8251	0.9012	0.6445
		5-6mer	0.8254	0.7707	0.8801	0.902	0.6547
	6mA_T.thermophile	3-mer	0.8864	0.9696	0.8033	0.9294	0.7535
		4-mer	0.8764	0.9596	0.7933	0.9394	0.7635
		5-mer	0.8799	0.9517	0.8082	0.9425	0.7678
		6-mer	0.8797	0.9607	0.7986	0.9436	0.7695
		3-4mer	0.8789	0.9514	0.8065	0.9408	0.766
		3-5mer	0.8795	0.9591	0.7999	0.9426	0.7688
		3-6mer	0.8804	0.9442	0.8167	0.9438	0.7671
		4-5mer	0.8809	0.9417	0.8202	0.943	0.7676
		4-6mer	0.8808	0.956	0.8055	0.9443	0.7703
		5-6mer	0.8818	0.9435	0.8202	0.9443	0.7695
	6mA_Tolypocladium	3-mer	0.7469	0.7987	0.6951	0.8275	0.4965
		4-mer	0.7472	0.7371	0.7371	0.8257	0.4945
		5-mer	0.7451	0.7946	0.6957	0.8252	0.4926
		6-mer	0.7694	0.7152	0.8236	0.8469	0.542
		3-4mer	0.6744	0.7999	0.5488	0.7575	0.3603
		3-5mer	0.7179	0.8135	0.6223	0.8035	0.444
		3-6mer	0.7771	0.7649	0.7892	0.85	0.5543
		4-5mer	0.7256	0.7614	0.6898	0.8022	0.4523
		4-6mer	0.7644	0.7466	0.7821	0.83	0.529
		5-6mer	0.7768	0.7407	0.8129	0.8553	0.555
	6mA_Xoc BLS256	3-mer	0.8706	0.8886	0.8527	0.9424	0.7417
		4-mer	0.8735	0.8784	0.8687	0.9391	0.7471
		5-mer	0.8686	0.8794	0.8578	0.9409	0.7374
		6-mer	0.8767	0.8865	0.867	0.9491	0.7536
		3-4mer	0.8749	0.8887	0.8612	0.9459	0.7501
		3-5mer	0.8753	0.8667	0.8839	0.9477	0.7508
		3-6mer	0.8817	0.8808	0.8827	0.9506	0.7634
		4-5mer	0.875	0.8784	0.8716	0.9446	0.75
		4-6mer	0.8785	0.893	0.8639	0.9496	0.7573
		5-6mer	0.8757	0.8993	0.8522	0.9476	0.7523

Table S11. Performance comparison with the 5mC methods on cancer cell line Encyclopedia (CCLE).

Model	ACC	SE	SP	AUC	MCC
iPromoter-5mC	0.9022	0.8777	0.9042	0.9570	0.5771
5mC_Pred	0.9180	0.8950	0.9200	0.9620	0.6250
BiLSTM-5mC	0.9303	0.8661	0.9374	0.9635	0.6384
iDNA-ABF	0.9353	0.7891	0.9477	0.9654	0.6323

Table S12. Performance of iPromoter-5mC in three human cell lines.

Cell Lines		ACC	SE	SP	AUC	MCC
**K562**	11bp	0.5164	0.6498	0.3831	0.5212	0.0341
	41bp	0.7113	0.7785	0.6441	0.7822	0.4264
	71bp	0.7177	0.8211	0.6144	0.7917	0.4451
	101bp	0.7121	0.8566	0.5676	0.7870	0.4431
**GM12878**	11bp	0.5176	0.6361	0.3991	0.5248	0.0362
	41bp	0.7330	0.6749	0.8990	0.7946	0.4940
	71bp	0.7370	0.9160	0.5580	0.8001	0.5077
	101bp	0.7372	0.9279	0.5465	0.7987	0.5133
**HepG2**	11bp	0.5302	0.5177	0.5427	0.5377	0.0604
	41bp	0.8221	0.8749	0.7693	0.8921	0.6478
	71bp	0.8454	0.8982	0.7926	0.9114	0.6947
	101bp	0.8583	0.9041	0.8125	0.9195	0.7196

Table S13. Training parameters of our model on 17 benchmark datasets.

Dataset	Batch_size	Learning_rate	Test ACC
5hmC_H.sapiens	64	0.0001	95.01
5hmC_M.musculus	64	0.0001	96.85
4mC_C.equisetifolia	32	0.0001	85.79
4mC_F.vesca	128	0.00005	85.24
4mC_S.cerevisiae	64	0.00005	72.30
4mC_Tolypocladium	64	0.0001	74.34
6mA_A.thaliana	64	0.00005	86.03
6mA_C.elegans	32	0.00005	91.38
6mA_C.equisetifolia	64	0.00005	73.99
6mA_D.melanogaster	64	0.0001	92.28
6mA_F.vesca	64	0.0001	94.13

6mA_H.sapiens	128	0.00005	91.04
6mA_R.chinensis	32	0.0001	86.29
6mA_S.cerevisiae	128	0.00005	82.78
6mA_T.thermophile	32	0.0001	88.04
6mA_Tolypocladium	64	0.0001	77.71
6mA_Xoc BLS256	64	0.0001	88.17

Table S14. Performance of our iDNA-ABF for the SNP classification.

Dataset	Model		ACC	SE	SP	AUC	MCC
SNP	iDNA-ABF	11bp	0.5975	0.755	0.44	0.575	0.2055
		41bp	0.5375	0.68	0.395	0.5337	0.0782
		71bp	0.5725	0.83	0.315	0.564	0.1692
		101bp	0.53	0.705	0.355	0.5046	0.0641

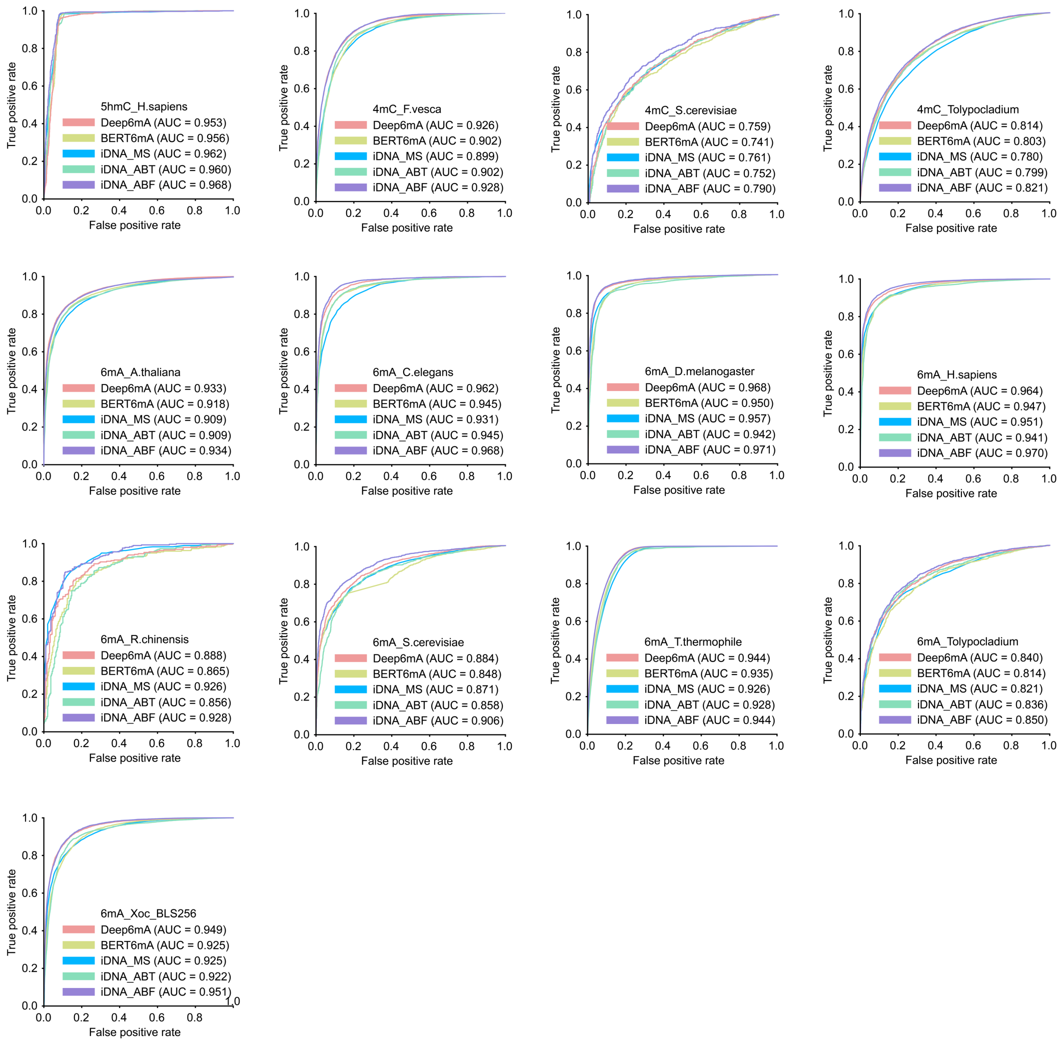

Figure S1. The ROC curves on remain benchmark datasets. *The datasets include*

5hmC_H.sapiens, 4mC_F.vesca, 4mC_S.cerevisiae, 4mC_Tolypocladium, 6mA_A.thaliana, 6mA_C.elegans, 6mA_D.melanogaster, 6mA_H.sapiens 6mA_R.chinensis, 6mA_S.cerevisiae, 6mA_T.thermophile, 6mA_Tolypocladium, and 6mA_Xoc BLS256.

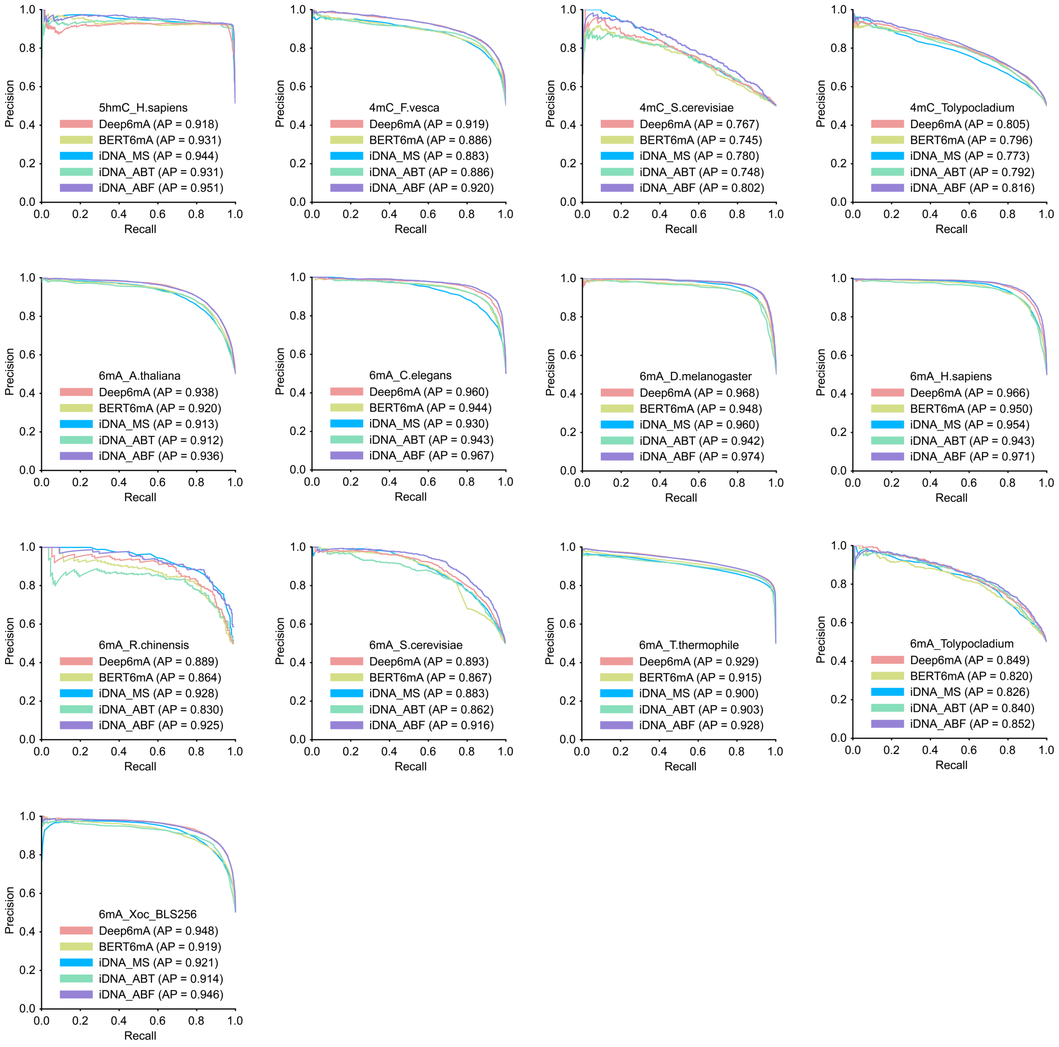

Figure S2. The PR curves on remain benchmark datasets. *The datasets include 5hmC_H.sapiens, 4mC_F.vesca, 4mC_S.cerevisiae, 4mC_Tolypocladium, 6mA_A.thaliana, 6mA_C.elegans, 6mA_D.melanogaster, 6mA_H.sapiens 6mA_R.chinensis, 6mA_S.cerevisiae, 6mA_T.thermophile, 6mA_Tolypocladium, and 6mA_Xoc BLS256.*

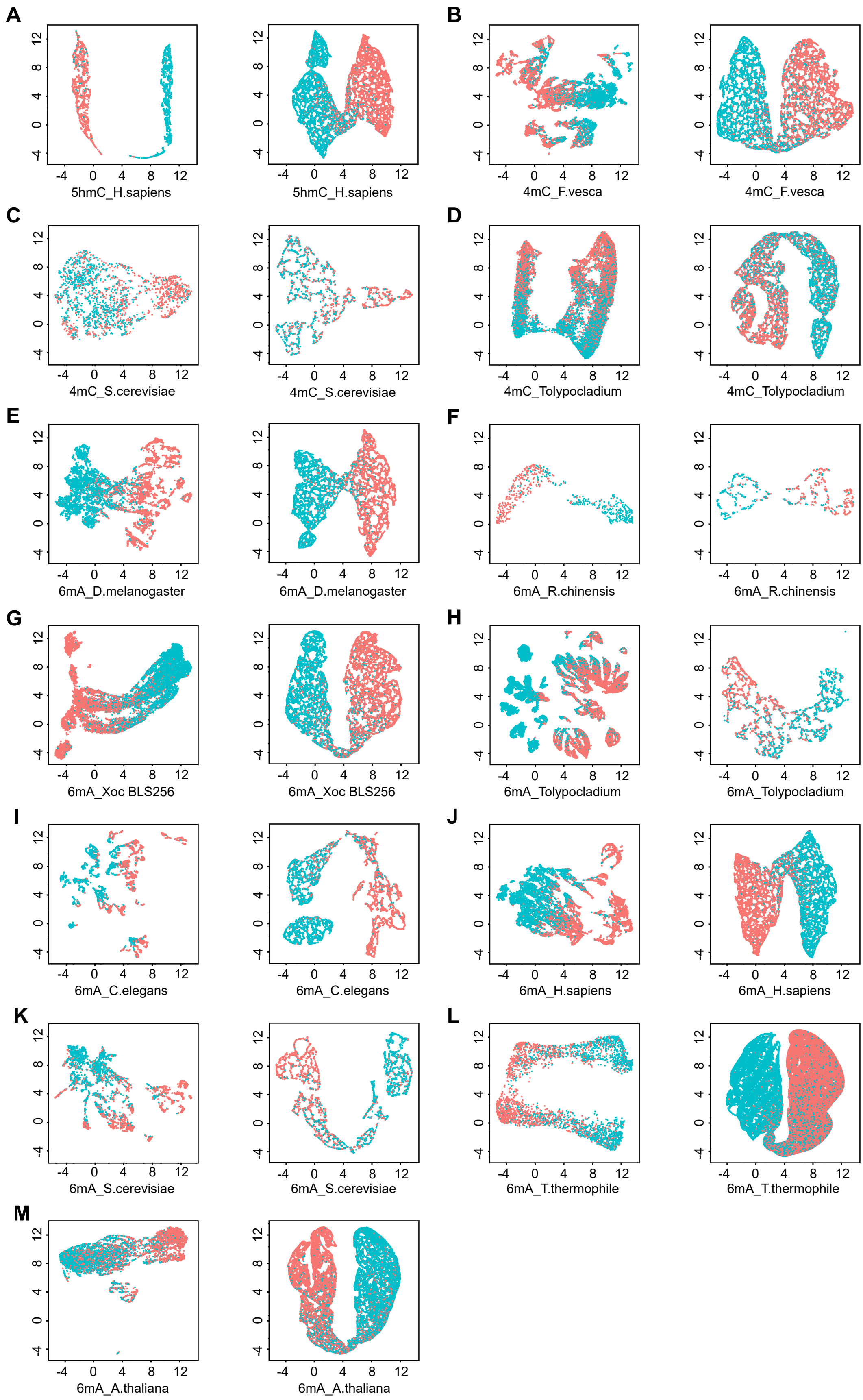

Figure S3. The UMAP visualization results on benchmark datasets. In each sub-

figure, the left part is the result of iDNA-ABF and the right part is the result of iDNA-ABT.

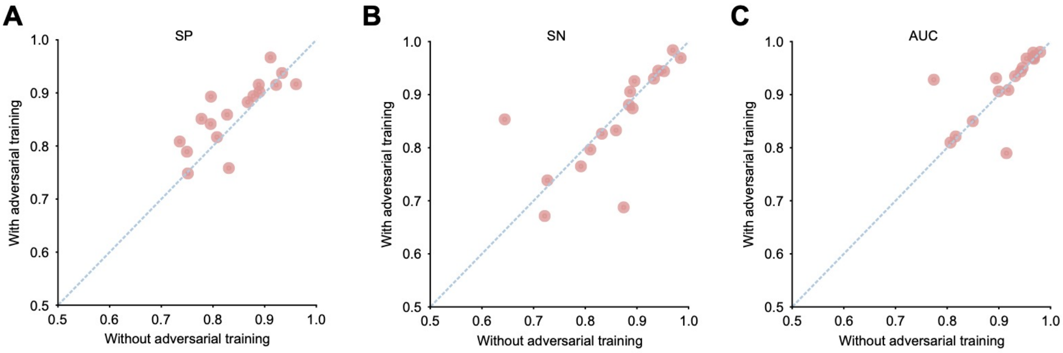

Figure S4. The SN, SP, and AUC of the models with and without adversarial training on 17 benchmark datasets with the independent test.

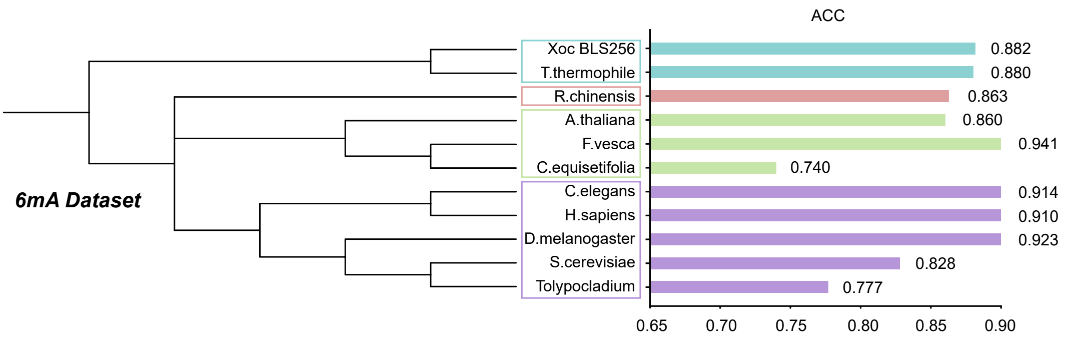

Figure S5. Taxonomy tree and accuracy for eleven species in 6mA dataset. Due to different scales of the datasets, the accuracy performance is not stable, but general trend and conclusion is similar to the conclusion in the main text.

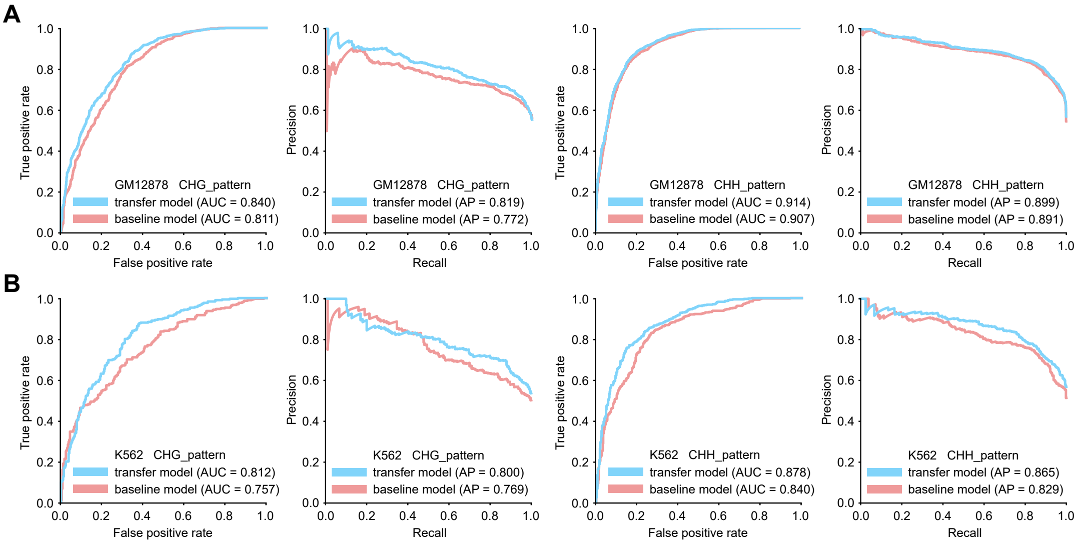

Figure S6. The ROC and PR curves of our baseline model and our model transferring from CpG pattern on the other two pattern datasets (i.e., CHG, CHH) in the cell line GM12878 and K562.

Supplementary methods

1. The multi-task model

In this model, we try to solve the problem of how to train a general model to predict more methylation types. So, we design a multi-task to balance the problems of how to find methylation site and how to distinguish methylation type. One task is just to make a binary classification on whether this site is DNA methylation. Another task is to distinguish which methylation type is. So, we choose three types of methylation in humans including 5hmC, 6mA, and 5mC. However, the result is lower than the most of model only trained on their own methylation type dataset. We think the reason may be that different methylation can have their own specific pattern and motif which is not easy to make accurate predictions.

Table S15. Performance of multi-task model.

	ACC	SN	SP	AUC	MCC
Multi-task	0.8695	0.8869	0.8521	0.9418	0.7395
6mA	0.9104	0.9057	0.9151	0.9104	0.8209
5mC	0.8579	0.8994	0.8165	0.8579	0.7184
5hmC	0.9501	0.9838	0.9164	0.9501	0.9022

2. The ChIP-seq data + sequence input model

In this model, we use ChIP-seq data as a four dimensional vector and use this vector to splice the representation vector extracted from our model. The final sequence representation is the combination of two vector. We use this representation vector to do a binary classification. To be mentioned, four dimension is composed of two Chip-seq data, including H3K4me3 and H3K36me3. In case of too much repeated value, we conclude one ChIP-seq data into just two value, which are calculated of the ChIP-seq average value by x-bp before this site and x-bp after this site. The experiment results can be seen in the Table S4.

3. The ChIP-seq data model

This model just feeds the ChIP-seq value of each bp into the DNN model. For example, if the sequence is 11 bp long, the model will feed a vector of 22 length that corresponds to two ChIP-seq reads. The experiment results can be seen in the Table S3.

4. The regression model built on ChIP-seq data

Among several ways to explore data fusion with ChIP-seq, in this model we further improve performance by fusing the loss function of the two tasks. The first task is a dichotomous task for methylation or not, and the other task is a regression task for ChIP values of the same sequence. The iDNA-ABF model in this paper is used for the binary classification task. The difference between the binary classification and the regression model lies in the final full connection layer. The regression task returns to a value, while the binary classification task is a two-dimensional vector.It is worth noting that the ChIP-seq data is the average obtained by H3K4me3 and H3K36me3, and many sequences correspond to an average of 0. In the process of signal loss calculation, the part where ChIP data is 0 is removed, and the mean value of the remaining loss is calculated to enhance the role of ChIP data.

Table S16. Performance of the regression model built on ChIP-seq data.

Cell Lines		ACC	SN	SP	AUC	MCC
GM12878	11bp	0.5000	1.0000	0.0000	0.5402	0.0000
	41bp	0.7494	0.8892	0.6095	0.8183	0.5195
	71bp	0.7370	0.9160	0.5580	0.8001	0.5077
	101bp	0.7615	0.8526	0.6704	0.8332	0.5319
K562	11bp	0.5055	0.0364	0.9746	0.5304	0.0318
	41bp	0.7355	0.8501	0.6209	0.8091	0.4839
	71bp	0.7177	0.8211	0.6144	0.7917	0.4451
	101bp	0.7386	0.9041	0.5732	0.8240	0.5058
HepG2	11bp	0.5339	0.6173	0.4504	0.5483	0.0687
	41bp	0.8287	0.8904	0.7670	0.8980	0.6625
	71bp	0.8454	0.8982	0.7926	0.9114	0.6947
	101bp	0.8653	0.9214	0.8093	0.9252	0.7353

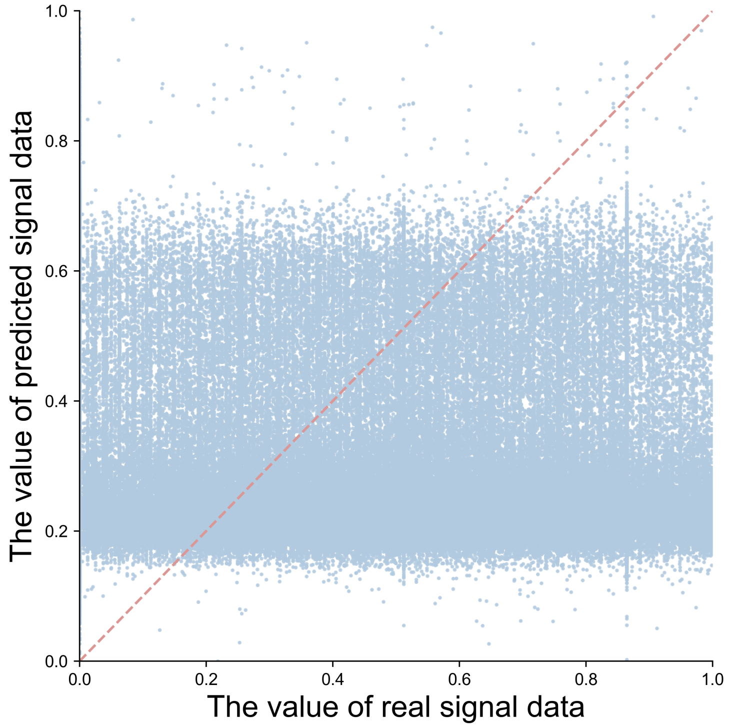

Figure S7. The regression result of signal prediction in the regression model built on ChIP-seq data.
